# Making clinical teaching visible—A time and motion study of hospital rounds in undergraduate medical teaching

**DOI:** 10.3389/fmed.2024.1377903

**Published:** 2024-08-21

**Authors:** Paddy Kilian, Nagam Alshehabi, Malek Othman, Anan Mahmoud, Leon du Preez, Nabil Zary, Samuel B. Ho

**Affiliations:** ^1^Mohammed Bin Rashid University of Medicine and Health Sciences (MBRU), Dubai, United Arab Emirates; ^2^Mediclinic Middle East, Dubai, United Arab Emirates

**Keywords:** clinical teaching quality, time management, medical undergraduate education, hospital ward rounds, clinical reasoning

## Abstract

**Background:**

Teaching medical students in the clinical setting is frequently perceived as a demanding commitment by attending physicians. There is a paucity of data measuring the duration and efficacy of teaching during clinical rounds.

**Aim:**

The aim of this study was to assess both the quantity and quality of clinical teaching time dedicated to medical students on hospital ward rounds.

**Methods:**

A cross-sectional direct structured observational study was conducted during the morning rounds of attending physicians involved in teaching undergraduate medical students at three different clinical facilities in three different specialties. A validated observational tool was used by four observers to record teaching time and quality indicators.

**Results:**

In terms of teaching duration, it was observed that 25% of the total morning round time was allocated to teaching. However, this measure varied widely between different physicians and specialties. As for teaching quality, actions categorized as active teaching by the teachers were observed in 19% of the interactions observed per round, while active learning by the students was observed in 17% of the interactions per round. Teacher high-cognition interactions were similarly observed in 23% of actions per round, while student high-cognition interactions occurred in 16% of actions per round. Internal Medicine tended to score higher than both Pediatrics and Surgery in terms of percentage teaching time as well as percentage of active teaching observed per round. Using liberal criteria, rounds characterized overall as predominantly active or high-cognition by both teachers and students were observed in only 21% of the total number of rounds observed.

**Conclusion:**

These results indicate that the percentage of teaching time during ward rounds is highly variable, and that round teaching generally consists of passive and low-cognition interactions. Future work is needed to train clinical faculty to achieve a desired level of teaching quality, and to determine if there are any changes in teaching time commitments and student outcomes.

## 1 Introduction

Undergraduate medical schools worldwide are facing an increasingly difficult task of equipping medical graduates with model knowledge, skills, and attributes in order to elevate the global healthcare standard. Achieving the balance between clinical services and teaching activities is complex, with several factors influencing the successful delivery of teaching, such as time constraints, clinical workload, administrative tasks, and both physician and student-specific factors (1). Notably, teaching medical students on hospital wards during the clinical years can be time consuming, requiring considerable faculty activity and dedication (2, 3). Quantifying the time spent teaching has been reported in a small number of studies using several methodologies, including direct observations (4, 5), video recording and analysis (6), time-motion studies (7–9), surveys and questionnaires (10, 11), and wearable technology such as real-time locating systems (12, 13).

The delivery of high quality clinical teaching during ward rounds is essential in cultivating competent and compassionate medical professionals (14). Medical students and trainees gain invaluable opportunities to observe and participate in the delivery of patient care, refine diagnostic skills and comprehend treatment decision-making in addition to learning communication skills, clinical reasoning and ethical principles (15). Several different methodologies have been used measure the quality of clinical teaching delivered including self-administered questionnaires (16), focus group discussions and other validated tools such as the Clinical Learning Environment, Supervision and Nurse Teacher (CLES + T) used in nursing education (17) and the bottom-up feedback Swiss System for Evaluation of Teaching Qualities (SwissSETQ) (18). Our study used direct structured observation to document the type of questions asked by the teachers and students, classifying these as high-cognitive or low-cognitive, in addition to observing the style of the teaching and learning, classifying it as either active or passive. These categories of quality have been used previously in the clinical teaching setting (7). Active teaching on ward rounds seeks to engage medical students in the learning process by using strategies that include interactive discussions, encouraging questions, hands-on learning and promoting clinical reasoning and decision making (19, 20). Passive teaching consists of relaying facts and principles or demonstrating to students without their active engagement or interaction, while passive learning involves students listening and observing without active participation (15, 21). Similarly, high-cognition refers to engaging students to demonstrate more complex thinking processes such as critical analysis and synthesis of concepts (22). For instance, the physician might ask the following of the student on the ward, “Given the patient's symptoms and history, what differential diagnoses are you considering and why?” In contrast, low-cognition teaching focuses on learning facts, memorizing, and repeating basic knowledge. Low-cognitive questioning of medical students on ward rounds involves questions that rely on basic facts recall and does not engage deeper level thinking or analysis, such as “What are the three most common causes of pneumonia?” (23). For the purposes of our study, we categorized good teaching quality as active teaching on the part of the teachers, and active learning on the part of the students, and as high-cognition actions on the part of the teacher and the student.

Analyzing both teaching time and quality metrics is vital in order to establish a baseline for understanding clinical teaching activities, informing actions aimed at improving the learning experience, and assisting in resource allocation decisions. This study focused on quantifying the actual time teaching and measuring the quality of these learning interactions as observed in routine hospital morning ward rounds. These hospital ward rounds are clinical service rounds when the physician team (usually the attending, designated nurse and hospitalists) meet and round from bed to bed to discuss each patient and make clinical decisions, allowing time to address any questions or concerns, update overnight information, and to ensure the healthcare team share a common understanding of the patient's care plan (24). Medical students who are undertaking their clinical clerkship rotation in a particular specialty will join in the ward rounds for that specialty. The teaching activities usually consist of a combination of bedside teaching and teaching outside the patient room.

The specific research objectives are: (1) To measure the quantity of undergraduate clinical teaching occurring in hospital morning ward rounds in a private hospital system across three different clinical disciplines (Internal Medicine, Pediatrics and Surgery). (2) To measure the quality of teaching based on categories of active vs. passive and high-cognition vs. low-cognition that occur during these clinical interactions.

## 2 Methods

The study consisted of direct structured observation of undergraduate teaching time and quality of hospital specialty-specific bedside ward rounds at three different private clinical tertiary hospital facilities affiliated with Mohammed Bin Rashid University of Medicine and Health Sciences (MBRU) in Dubai, UAE. The three hospitals form part of the Mediclinic Middle East Group and are approved by the local regulatory body, Dubai Health Authority, as clinical training facilities. These are Mediclinic City Hospital (MCIT), Mediclinic Parkview Hospital (MPAR) and Mediclinic Welcare Hospital (MWEL).

### 2.1 Research design

Our study is a cross-sectional observational study that uses quantitative methods to quantify teaching time and describe the quality of teaching during morning rounds.

Particularly, we chose a time-and-motion approach, which has been shown to provide more reliable information about events, with greater precision regarding the timing, duration and frequency to accurately describe large scale social events (25).

### 2.2 Participants

The studied population included 58 fourth year undergraduate students, 25 adjunct physician consultants, and 17 affiliated hospitalists across three tertiary-care hospitals (MCIT, MPAR and MWEL). These hospitals are located in Dubai, United Arab Emirates. The medical undergraduate students are enrolled in a 6-year curriculum and are annually assigned to cohort rotating groups for the duration of clinical academic years (years 4–6), while affiliated clinicians are assigned by the Mediclinic discipline coordinators to the teaching sessions. During their 4th year of study, medical students from MBRU rotate through five core specialties, namely Internal Medicine, Surgery, Pediatrics, Family Medicine and Behavioral Medicine in set 8-week blocks. Apart from Behavioral Medicine, all the above specialty-rotations take part in the Mediclinic Dubai facilities. The consultant physicians (attendings) and hospitalists are employed by Mediclinic but hold adjunct MBRU faculty status and form part of the clinical teaching faculty.

Inclusion criteria for observable rounds included morning ward rounds with at least a single teacher-student unit (comprising a minimum of one consultant and a minimum of one year-4 medical student) under any of the three disciplines (Internal Medicine, Pediatrics or Surgery) in any of the three observation hospitals (MCIT, MPAR or MWEL).

Exclusion criteria included hospitalist-led rounds, rounds under specialized disciplines (i.e., Ophthalmology, Intensive Care Unit, or Orthopedics), year-5 or year-6 students rounds or evening rounds. In addition, the Anesthesia component of the Surgery core course was excluded from observation due to lack of identifiable rounds.

The study was approved by the Mediclinic Middle East (MCME) institutional review committee (MCME.CR.270.MCIT.2022) and the Dubai Health Authority (DSREC Permit Ref No: DSREC-01/2023_15). Informed consent was also taken from every observed student, hospitalist and consultant using a digital form alongside verbal explanation of the study, the nature of data collection and the anonymity of all data collected. No case of participation refusal or withdrawal was observed.

### 2.3 Instruments

Observed rounds were analyzed using a modified version of the Structured Learning Observation Tool (SLOT) developed by Young et al. (7) (Table 1). As described previously, the original SLOT content was developed based on the six components of Bloom and Krathwohl's taxonomy, including knowledge, comprehension, application, analysis, synthesis and evaluation (26) and components of active and passive learning (27). The original SLOT was published in 2009 is composed of 17 different actions comprising groups of variables corresponding to different teaching and learning types (active vs. passive, low-cognition vs. high-cognition) as well as barriers to learning and teaching (7). There are no current studies that have used the same described SLOT to date to evaluate the quality of teaching on ward rounds, although a similar recent prospective observational study of Internal Medicine morning reports focused on the quantity and type of comments made by the attending, but not those made by the students or trainees. Comments were divided into teaching and non-teaching comments, but did not differentiate between high-cognitive or low-cognitive teaching or learning (28). In addition to modifying the SLOT, we included in the instrument components of commonly used clinical teaching strategies, including the “One Minute Preceptor” (29) and the “SNAPPS” technique (30, 31) that were currently being used in faculty development programs in the medical school (Table 1).

**Table 1:** The modified SLOT survey.

**Time and motion of undergraduate education—assessors' questionnaire**		
**1**		Consent taken from students and physicians?
**2**		Assessor's name
**3**		Date of the assessment
**4**		Location of the assessment
**5**		Specialty assessed
**6**		What participants were present during the round?
**7**		Round's start time
**8**		Round's end time
**9**		Teaching time focused on students
**10**		Describe patients and general interactions observed during the round
**11**	02a	Teacher asks closed question**—**no reference to prior knowledge
**12**	03a	Teacher asks closed question**—**draws on prior knowledge
**13**	04a	Teacher asks open ended question**—**no reference to prior knowledge
**14**	05a	Teacher asks open ended question**—**draws on prior knowledge
**15**	06a	Teacher asks student to apply clinical reasoning skills
**16**	07a	Teacher shares clinical experience
**17**	08a	Teacher uses think aloud strategy or outlines/summarizes
**18**	09a	Teacher demonstrates procedure
**19**	12a	Teacher refers to resources (print/online) or labels photos/diagrams
**20**	13a	Teacher shares knowledge in a didactic manner
**21**		Hospitalist participates in teaching or demonstration
**22**	02b	Student asks closed question**—**no reference to prior knowledge
**23**	03b	Student asks closed question**—**draws on prior knowledge
**24**	04b	Student asks open ended question**—**no reference to prior knowledge
**25**	05b	Student asks open ended question**—**draws on prior knowledge
**26**	06b	Student asks another student to apply clinical reasoning skills
**27**	07b	Student shares clinical experience
**28**	08b	Student uses think aloud strategy or outlines/summarizes
**29**	09b	Student demonstrates procedure
**30**	12b	Student refers to resources (print/online) or labels photos/diagrams
**31**	13b	Student shares knowledge in a didactic manner
**32**	10	Student recalls descriptive knowledge
**33**	11	Student demonstrates understanding and use of higher-level cognitive functioning
**34**		Hospitalist asks question as above
**Specific teaching tools: One Minute Preceptor elements**		
**Mark each time observed and describe the interaction**		
**35**		Summarize and ask student for a commitment
**36**		Probe understanding “Why do you think this?”
**37**		Reinforce what is done well
**38**		Teach clinical pearl or general principles
**39**		Correct errors**—**“sandwich style”
**40**	S	Ask student to summarize
**41**	*N*	Narrow differential
**42**	A	Analyze reasons why
**43**	P	Preceptor probes student
**44**	P	Plan
**45**	S	Identify topic to study

As described previously, the SLOT categorizes active teaching and learning to include open-ended, critical reasoning questions and think-aloud strategies. Passive teaching and learning consisted of closed-ended questions, reference to diagrams, and didactic teaching. High-cognitive teaching and learning includes both open-ended questioning and application of critical reasoning skills, whereas low-cognitive teaching and learning includes close-ended questions and recall of information (7).

Inter-observer reliability was verified by data collected in control sessions having two independent observers in the same group (Table 2) and was shown to indicate a significant level of inter-observer agreement across all control sessions (*P* < 0.05).

**Table 2:** Interobserver reliability measures for four control sessions.

**Control session**	**Interobserver reliability measures**		
	**Assessors**	**Interobserver reliability**	***P*** **-value**
1	1	0.995	0.001
	2		
2	1	0.997	0.001
	2		
3	2	0.936	0.001
	3		
4	4	0.985	0.001
	1		

### 2.4 Procedure

A training session of the observers took place on 29th March 2023 during which the components of the modified SLOT form were explained with examples given. The sampling technique consisted of each of the four trained observers (one faculty and three year-6 students) attending a pre-set number of rounds at random days within the time-period between 18th April 2023 and 21st June 2023 depending on the observers' availability. Notably, the data collection period coincided with the last two clinical rotations of the academic year.

Observers took explicit oral consent before the beginning of the rounds and then often completed the written consent form after the rounds, while giving the participants the opportunity to withdraw at any point of the observation. Although we have observed a consistently low percentage of teaching time, the explicit consent prior to the round might have biased the teachers to allocate more time to undergraduate teaching during the round and thus the explicit consent at the beginning of the round should be noted as a possible confounding factor.

Direct observation of the timings of clinical interactions during hospital ward rounds was performed using a modified SLOT form digitized on SurveyMonkey^®^ (http://www.surveymonkey.com), a robust online data collection tool (32), to allow simplified input of all the components examined on portal devices. Similar studies assessing the quality of clinical teaching have utilized SurveyMonkey^®^ as the data collection tool (33–35). Appointed observers noted the interactions between clinicians, students, and hospitalists as non-participating members of the clinical group, and were instructed to avoid engagement with the team in terms of answering questions or contributing to the teaching discussions.

### 2.5 Data analysis

#### 2.5.1 Data protection

After all anonymized data was inputted into the digitalized SLOT on SurveyMonkey^®^, the raw data was extracted as an excel file by a single designated investigator.

#### 2.5.2 Data cleaning and coding

Round time was calculated from the observed round start and end time in minutes. Subsequently, the observed teaching time durations in each round were used to calculate the average percentage teaching time.

As for teaching quality variables (active vs. passive learning and teaching; high-cognition vs. low-cognition learning and teaching), the component questions devised by Young et al. (7) were combined to represent each quality variable. Afterwards, the number and percentage of active, passive, high- and low-cognition actions were calculated for both teacher and student arms in each individual round; allowing the calculation of the average percentage of active and high-cognition actions.

### 2.6 Framework for the qualitative analysis

Directly observed measures of teaching quality defined by an active vs. passive spectrum and a high- vs. low-cognitive spectrum are used as factors for defining the quality of clinical teaching. This framework is used to map a range of teaching types from Active/High-Cognition to Passive/Low-Cognition. For this framework we categorized each of the 24 teaching rounds according to overall active vs. passive teaching/learning and high-cognition vs. low-cognition based on whether a significant percentage (arbitrarily defined as 50% or above or more liberally as 20% or above) of teacher and student observations were active vs. passive or high- vs. low-cognition. These thresholds have produced four possible categories of all rounds observed on the Active-Passive spectrum: (1) Active Teacher–Active Student; (2) Active Teacher–Passive Student; (3) Passive Teacher–Active Student and (4) Passive Teacher–Passive Student. Additionally, four further categories of all rounds were produced using the High-Low Cognition spectrum: (1) High-Cognition Teacher–High-Cognition Student; (2) High-Cognition Teacher–Low-Cognition Student; (3) Low-Cognition Teacher–High-Cognition Student and (4) Low-Cognition Teacher–Low-Cognition Student.

### 2.7 Statistical analysis

Descriptive statistics were used to calculate the means, medians, and Standard Error Measures (SEM) for the teaching duration parameters as well as teaching quality variables. Descriptive statistics were chosen as they were deemed to play a vital role in understanding and evaluating both the quantity and quality of clinical teaching during the hospital ward rounds by providing a concise summary of key metrics, especially when these metrics have been attained via observation (36). In addition, outliers were included in the final analysis. This was followed by ascertaining inter-observer reliability and performing two-tailed ANOVA tests to compare the parameters of teaching duration and quality across specialties.

## 3 Results

In total, 28 rounds were observed, including four control rounds where two observers were present. The total observation time amounted to 19 h and 36 min. Observers reported rounding on 93 patients (with a mean of 3.9 patients per round) and a total of 58 year-4 students observed (some students were repeated, with a mean of 2.4 students per round), majority of which are female reflecting the higher proportion of female students in MBRU. As for faculty, a total of 25 consultants were observed (with a mean of 1 consultant per round). Of note, 19/24 rounds observed (79.2%) included a hospitalist and 10/24 rounds observed (41.7%) had at least one absent student.

### 3.1 Quantity of teaching time

**Figure 1:**
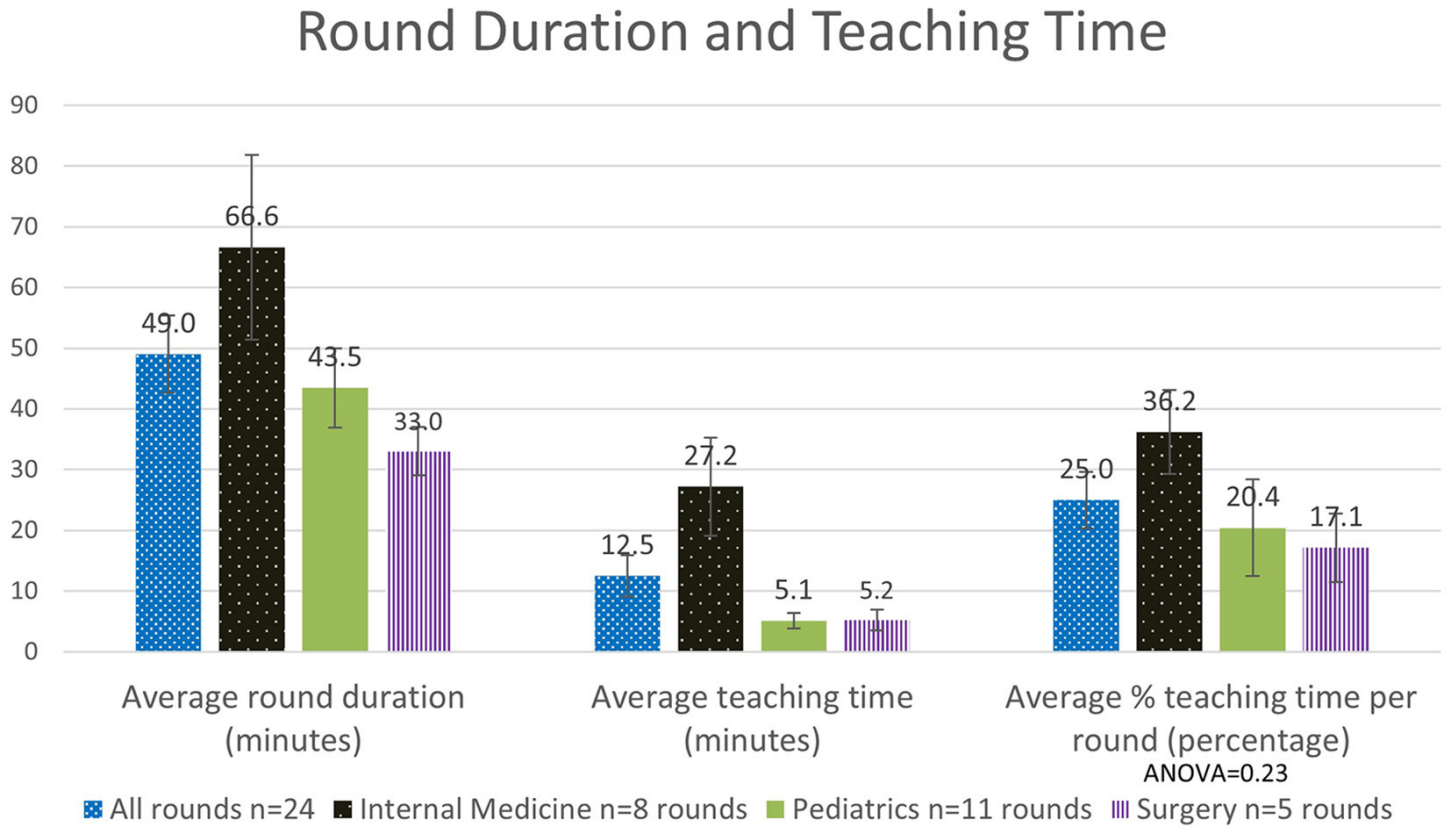
Analysis of the quantity of time spent teaching, as measured overall and across specialties (*n*: number).

The overall average hospital round duration across all 24 round observations in all specialties was 49.0 ± 6.3 min SEM, with an average teaching time of ~12.5 ± 3.4 min SEM. This equates to an observed average percentage time spent teaching of 25.0 ± 4.6% SEM. For each specialty the average round duration varied for Surgery (33.0 ± 4.0 min SEM), Pediatrics (43.5 ± 6.6 min SEM), and Internal Medicine (66.6 ± 15.2 min SEM). The average percentage teaching time per round was highest for Internal Medicine (36.2 ± 6.9% SEM), followed by Pediatrics (20.4 ± 8.0% SEM) and Surgery (17.1 ± 5.6% SEM; Figure 1).

### 3.2 Quality of teaching time

#### 3.2.1 The percentage of time teaching engaged in active teaching vs. passive learning

**Figure 2:**
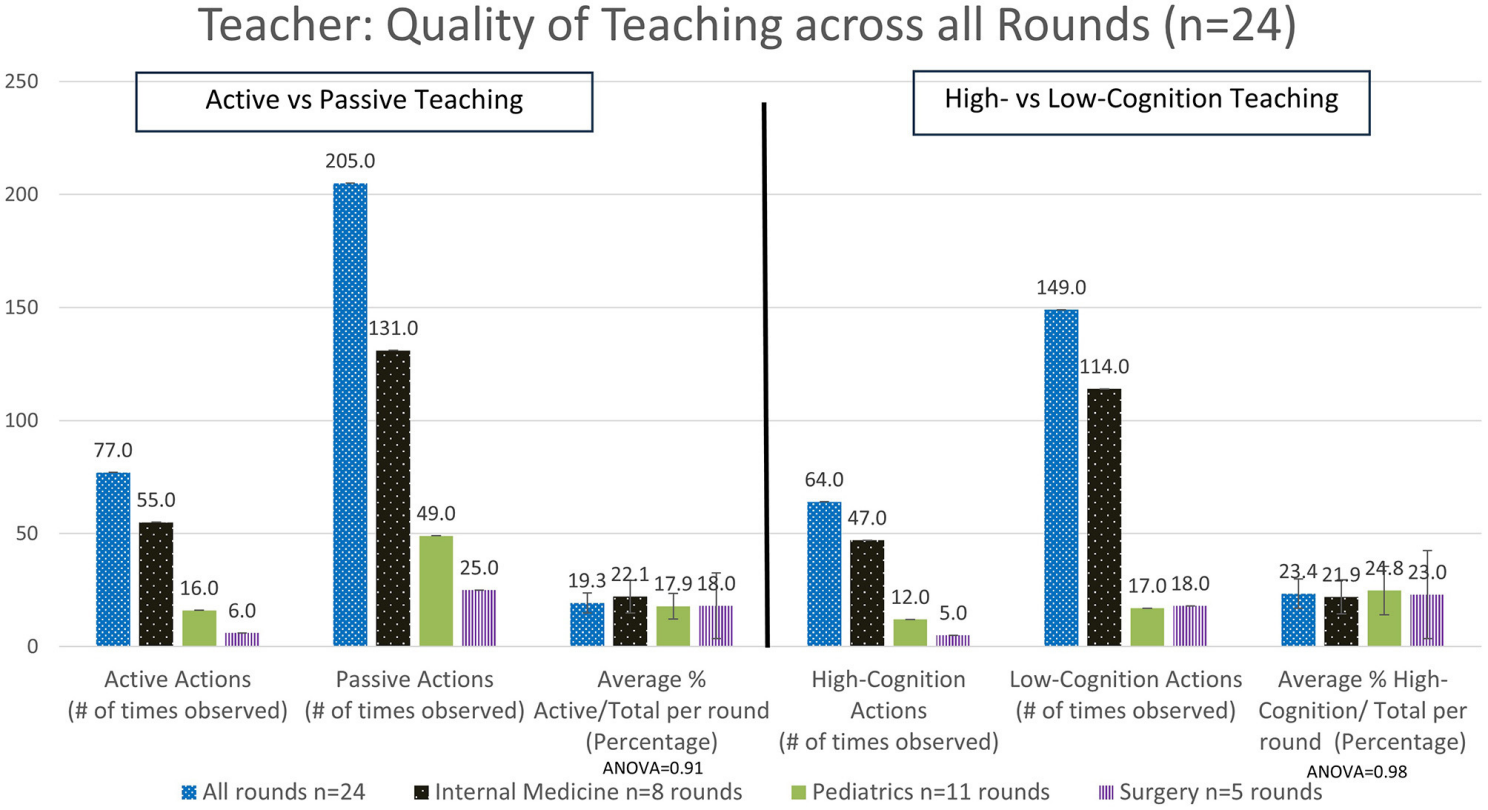
Analysis of the quality of the time spent teaching as delivered by the teacher - active vs. passive actions and high- vs. low-cognitive actions (#: number, *n*: number).

**Figure 3:**
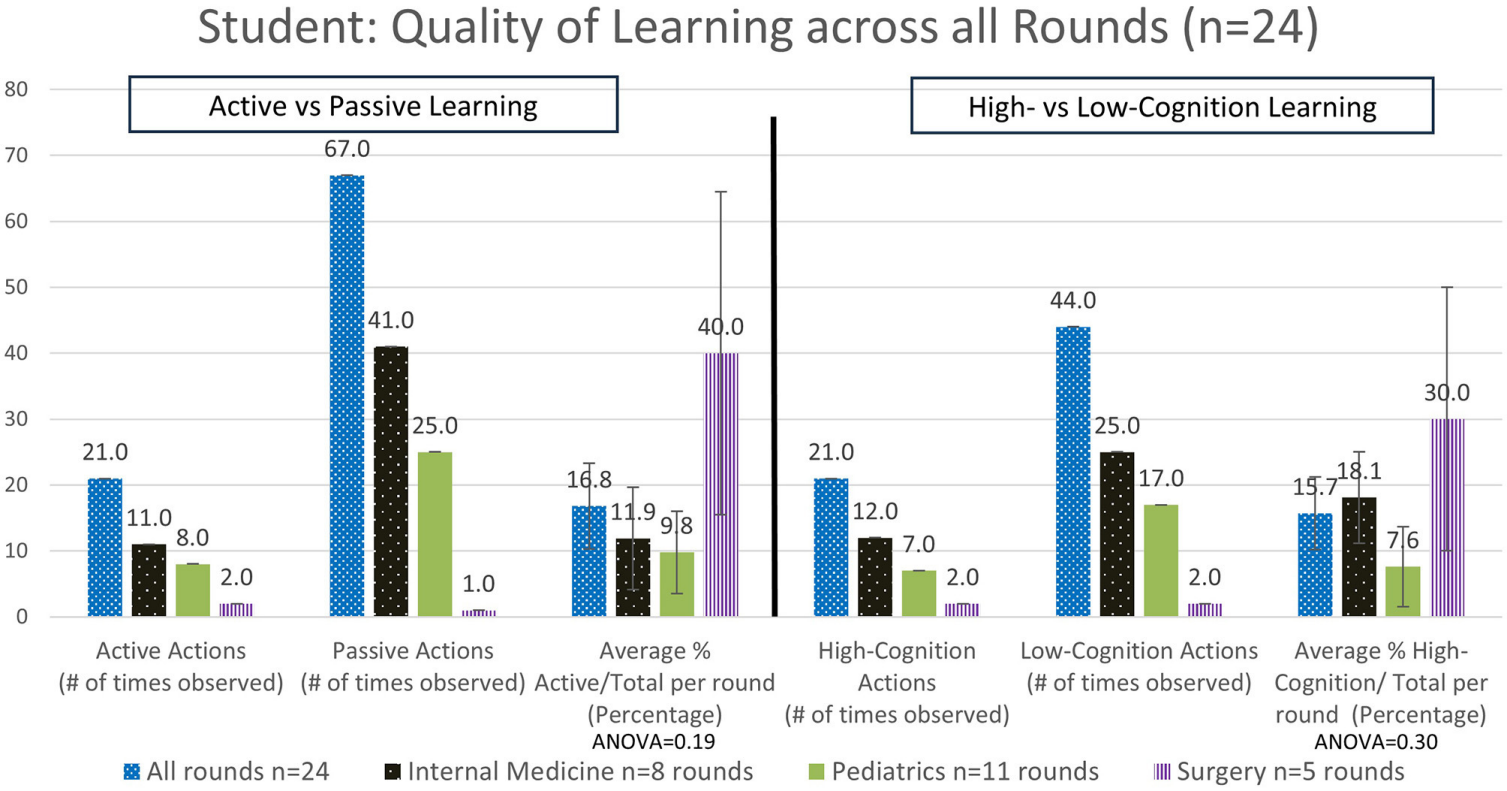
Analysis of the quality of the time spent learning by the students—active vs. passive actions and high- vs. low-cognitive actions (#: number, *n*: number).

Overall, for all rounds the teachers were observed to demonstrate active teaching in 19.3 ± 4.4% SEM of teaching interactions (Figure 2), and the students were observed to demonstrate active learning in 16.8 ± 6.5% SEM of interactions (Figure 3). Again, this varied by specialty, with the highest demonstration of active teaching by teachers observed in Internal Medicine, followed by Pediatrics and then Surgery. In contrast, the percentage active learning by students was highest in Surgery, followed by Internal Medicine and then Pediatrics (Figure 3). However, note that the number of observations in Surgery was very small.

#### 3.2.2 The percentage of time teaching engaged in high-cognition teacher actions and high-cognition student actions

Categorizing teaching in terms of cognition overall, we observed teachers delivered high cognition in 23.4 ± 6.5% SEM of interactions (Figure 2). There were no significant differences observed when comparing high-cognitive teacher actions across the different specialties. In contrast, a trend for high-cognition student actions was observed to be higher in Surgery, followed by Internal Medicine and then Pediatrics (Figure 3). Ward round teaching techniques include the widely recognized One Minute Preceptor (Table 3) and SNAPPS methods (Table 4), yet we rarely observed elements of these teaching techniques being used.

**Table 3:** Observations of the elements of the One-Minute Preceptor Technique.

**Where**	**The One-Minute Preceptor Technique (OMP)**					**Number**
**Location of observations**	**Get a commitment**	**Probe for supporting evidence**	**Teach general rules**	**Reinforce what was done right**	**Correct errors**	**Times complete technique was observed**
All rounds (*n* = 24)	31	8	35	2	1	0
Int. medicine (*n* = 8)	31	7	30	0	1	0
Pediatric (*n* = 11)	0	1	4	2	0	0
Surgery (*n* = 5)	0	0	1	0	0	0

**Table 4:** Observations of the elements of the SNAPPS technique.

**Where**	**The SNAPPS technique**						**Number**
**Location of observations**	**S Summarize the case**	**N Narrow differential**	**A Analyze differential**	**P Probe preceptor**	**P Plan management**	**S Select an issue for self-directed learning**	**No. of times when the complete technique was observed**
All rounds (*n* = 24)	4	10	7	15	3	5	0
Int. medicine (*n* = 8)	4	9	5	14	2	3	0
Pediatric (*n* = 11)	0	0	1	0	0	2	0
Surgery (*n* = 5)	0	1	1	1	1	0	0

#### 3.2.3 Characterization of individual hospital round teaching quality

**Figure 4:**
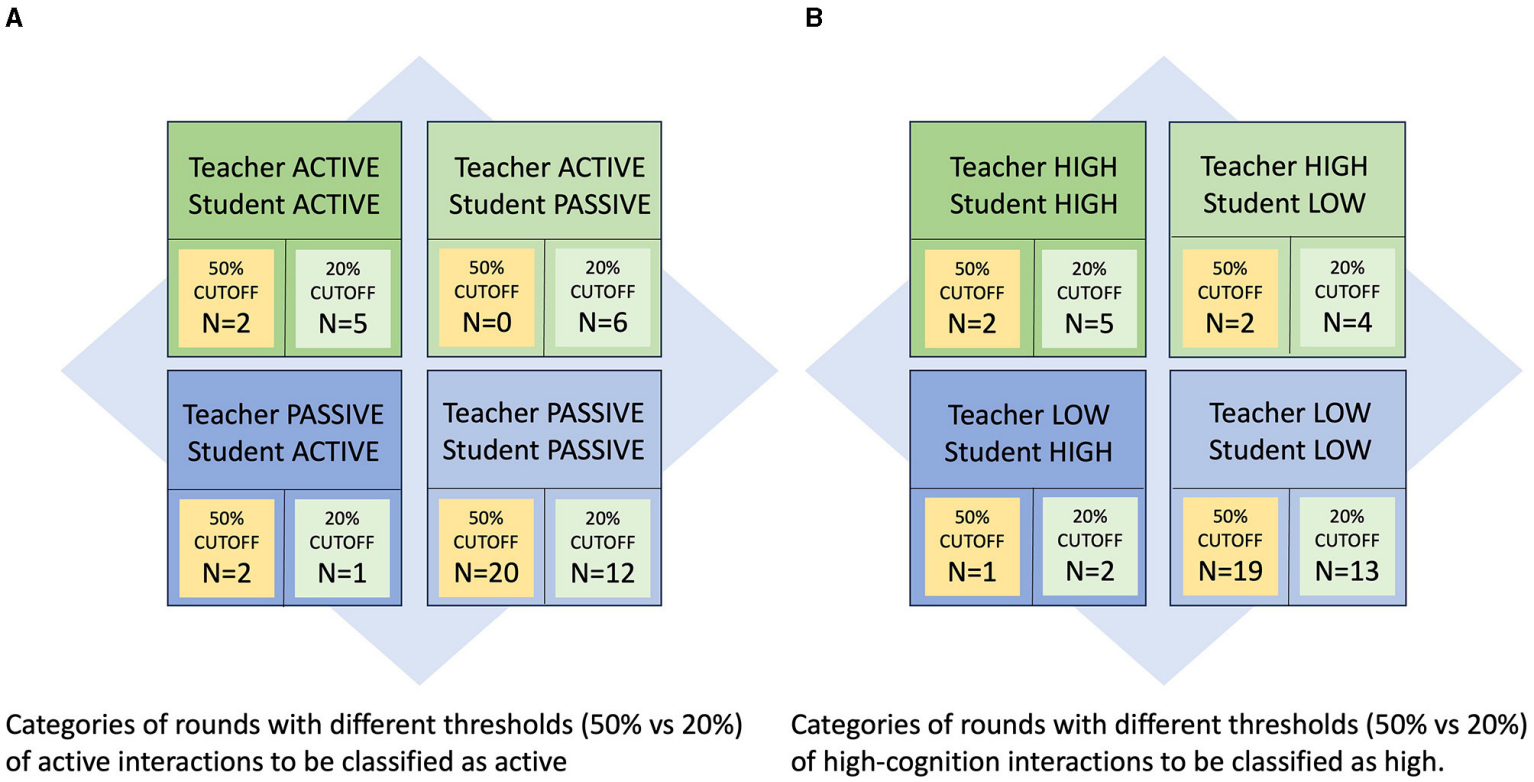
**(A)** Characterization of individual hospital round teaching quality using the proposed taxonomy (using a threshold of at least 50%; *n*: number). **(B)** Characterization of individual hospital round teaching quality using the proposed taxonomy (using a threshold of at least 20%; *n*: number).

We categorized each of the 24 teaching rounds according to overall active vs. passive teaching/learning actions and high-cognition vs. low-cognition actions based on whether a significant percentage of teacher and student observations were active vs. passive or high- vs. low-cognition. This resulted in a very few number of rounds that were found to be active teaching/learning or high-cognition. If we arbitrarily defined active teaching/learning and high-cognition as any rounds with 50% or above interactions meeting these criteria (Figure 4), then we found that the majority of rounds were classified as passive teacher/passive student in 20/24 (83%) of rounds, and classified as low-cognition teacher/low-cognition student in 19/24 (79%) of rounds. Alternatively, if we used the more liberal definition of active teaching/learning and high-cognition as any rounds with 20% or above interactions meeting these criteria (Figure 4), then the majority of rounds were still considered as passive teacher/passive student in 12/24 (50%) and teacher low-cognition/student low-cognition in 13/24 (54%); and considered as active for teacher/student learning and high-cognition for teachers/students in only 5/24 (21%) of rounds.

## 4 Discussion

### 4.1 Quantity of teaching time on ward rounds

In this active observational pilot study of teaching time and quality of inpatient ward rounds, we observed that approximately 25% of the time overall was used to address students. This varied widely between different physicians and specialties, as indicated by the wide standard deviations observed in all measurements. There are a limited number of studies that have objectively quantitated clinical teaching time in inpatient or outpatient settings using direct structured observation. Notably, a recent study using time-motion methodology was used to actively observe activities during the morning rounds and found that 18.2% of total rounding time was dedicated to bedside teaching (9), while a time motion study on internal medicine trainees in 2019 noted that only about 12% (24 min out of an average of 204 min on rounds daily) was spent on teaching activities per round (8). Another observational study focusing on bedside teaching in an intensive care unit noted that attending physicians spent 12% of the total rounding time teaching (17 min per day) (4). Other studies have utilized surveys and detailed questionnaires to quantify the time spent teaching on ward rounds, often with results showing minimal time spent teaching at the bedside (10, 11). Khan et al. (3) used an extensive questionnaire and found that 25% of ward rounds across various specialties at an academic center in Saudi Arabia were devoted to teaching. An indirect study focusing on time spent teaching noted the mean for outpatient teaching time was 0.65 h per clinic half day, while allotting 1.1 h per full day of inpatient time (37). These values fall within a similar range of time spent teaching compared to the 25% we observed in our study.

There are a number of factors that reduce the time available for supervision and active teaching during ward rounds, such as workload of the physicians (38, 39), while time constraints have often been highlighted as challenges to fully utilizing the teaching and learning benefits of ward rounds (10, 15). Several ward-rounding teaching models have been proposed in an effort to introduce a more efficient way of rounding that allows more time for teaching, such as the NET Rounding Intervention (40).

### 4.2 Quality of teaching time on ward rounds

For the purpose of the study, we categorized teaching quality as active teaching on the part of the teachers, and active learning on the part of the students, and as high-cognition actions on the part of the teacher and the student. Active teaching was observed in <20% of teaching interactions overall, as was a similar minority for high-cognitive actions on both the teacher and the student. It was noted that Internal Medicine tended to be higher than Pediatrics and Surgery in terms of percentage teaching time during morning ward rounds and in terms of percentage of active teaching and learning observed, however the small number of individual rounds observed in each specialty limits the accuracy of these results. Determinants of high-quality teaching were categorized as active teaching with high-cognition on the part of the teachers (19.3%−23.4% of interactions), and active learning with high-cognition on the part of the students (16.8%−15.7% of interactions). When the entire inpatient ward round episode is categorized as active learning or high-cognition on the part of teacher and student, only 21% of rounds could be considered high quality given the most liberal interpretation (Figure 4). With a stricter interpretation only 8% of rounds could be considered high quality (Figure 4).

Despite the geographic and temporal differences, these results were very similar to the study of Young et al. (7), using the same structured learning observation tool in a sample of 40 students in the final year of a graduate-entry program at the University of Queensland, Australia. They observed a predominance of passive and low-level cognitive actions during bedside learning interactions. Specifically, active teaching and learning was observed only 17.5% of the time and higher cognitive learning was observed only 5.8% of the time during a typical bedside teaching session (7). High-cognitive questioning by clinical educators promotes critical reasoning and this significantly enhances students' abilities to think critically and analyze their reasoning (41). Skillful questioning on the part of the clinical educator can improve the quality of clinical teaching on ward rounds by ensuring the content is relevant to the student. It allows the teacher to gauge the foundation of knowledge and helps the student to self-assess (42). Closed questioning by the teacher, on the other hand, with a focus more on clinical content and factual recall, as seen in the direct structured observation of pediatric trainees on consultant-led ward rounds, provided limited opportunities for clinical reasoning (43). Understanding the determinants of highly rated teaching qualities in clinical teachers and the perceptions of students can guide faculty development. Of note, teacher's interpersonal and communication skills and the importance of excellent role-modeling and communication have been highlighted (44) and similarly, good communication skills and a calm and non-humiliating demeanor were identified by trainees as most important qualities of a good teacher (45). One study found that teaching self-efficacy to a clinician educator greatly improved the quality of the teaching at the bedside, whereby teachers are equipped to handle the learning environment and students (such as handling new teaching methods or managing a learner experiencing difficulties) (46). Improving the quality of teaching on a ward round from a medical student's perspective includes actions such as getting the student involved, have them do a pre-round, take the patient's history and examine, then present the case, if asked to stay outside give them blood or imaging results to interpret and then present, and once patients have been seen, give them tasks to do, such as venipuncture, thus involving the student more in the active learning during ward rounds (47). Another method is teaching students illness scripts to help them formulate clinical questions while on the ward rounds and improve their clinical reasoning (48). Physicians felt that a “good” student should be interested in learning and communicate effectively with the teacher (15), while empowering the medical student to use critical thinking skills and clinical reasoning on ward rounds has been seen as essential (49). Quality can be improved by coaching the students, simulating ward rounds, teaching them how to ask questions, allowing them to be “invited and involved” and feel accepted as members of the team (1, 50). Thus both the student and the teacher play an important role in creating a successful high quality teaching experience (51).

### 4.3 Developing a taxonomy of clinical teaching

Our results suggest that clinical teaching time modified by a quality measure as reflected by the framework for analysis of clinical teaching would be an example of an accurate or fair conceptual model to serve as a benchmark for the evaluation of clinical teaching effort. It could assist with resource allocation for example, with possibly fewer resources required for passive and low-cognition activities compared to those needed for active and high-cognitive teaching. Additionally, categorizing teaching rounds in this way allows for specific feedback to the teachers and students in terms of the type of teaching that would be more desirable. Furthermore, refinement of the teaching taxonomy can be used to clarify and target the goals of particular clinical teaching interactions. For example, teaching that falls into the active teaching/low-cognitive category may be preferred with early clinical learners and could include demonstrations of physical exam and knowledge-based instruction. Teaching that is in the active teaching/high-cognition category would focus on challenging students to use and demonstrate clinical reasoning, which is appropriate for more advanced clinical learners.

Further research is also needed to determine if there are any differences in percentage of teaching time required for active and high-cognition teaching/learning. The difference between active and passive teaching may not entail any difference in time spent teaching. For example, when leaving a patient encounter, the teacher could simply ask “what do you think is going on with this patient?”, rather than “please list three most common causes of pneumonia?” This is an active and high-cognition teaching style that is incorporated into the widely used One Minute Preceptor or SNAPPS methods to use clinical teaching time efficiently. Despite many of the physicians being exposed to these concepts on prior faculty development lectures, we did not observe any instance in which these were consistently used. This points out the fact that active and high-cognition teaching requires the teacher to be knowledgeable about medical education in the clinical environment, and this could be a focus of targeted faculty development and ongoing individual observation and feedback of clinical teaching. Future work will be to develop faculty development to further refine and specify the desired clinical teaching taxonomy, and to determine which type of teaching results in better learning outcomes for students.

Our observation that most hospital rounds involve passive teaching and low-cognition actions on the part of both teachers and students indicates that efforts are warranted to try to enhance the quality of teaching during hospital rounds. In addition to promoting the use of techniques such as One Minute Preceptor and SNAPPs (52), several other teaching models have been developed specifically for bedside teaching, all with the goal of optimizing the clinical teaching and learning environment. These include, among others, the MiPlan model, the COX model, the Microskills of Teaching model and the Meeting to Meeting model (53, 54). With regards to addressing students' needs and problems in specific learning systems, surveys and frequent evaluations provide a reliable way to assess these issues. Buchanan et al. (55) engaged internal medicine residents in the improvement of rounds efficiency and teaching in a university hospital. Problems revealed by baseline surveys, were later fixed in a quality improvement project. Post-implementation survey results showed high overall satisfaction, perceived efficiency of rounds and preserved quality of education among residents.

### 4.4 Limitations

Literature searches have indicated some discrepancies using the time-activity approach as a vehicle to analyze the time spent teaching. One of the limitations is the potential for observer bias, as is inherent in all observation studies. To mitigate this bias, we used primarily graduated medical students as observers, which may have been less intimidating for the faculty. While using multiple observers introduces the possibility of errors, we found satisfactory inter-observer agreement when comparing different observers for overall teaching time in the same session. Another way to overcome this could be having a single researcher to time the actions of the student and preceptor and to record every activity. Additionally, this study is limited by the number of observations in the different specialties, which needs to be considered when interpreting the data. Further work will be required to modify and validate the structured observational tool and build the teaching taxonomies proposed.

## Data availability statement

The raw data supporting the conclusions of this article will be made available by the authors, without undue reservation.

## Ethics statement

The studies involving humans were approved by Mediclinic Middle East IRB and Mohammed Bin Rashid University of Medicine and Health Sciences IRB. The studies were conducted in accordance with the local legislation and institutional requirements. The participants provided their digital informed consent to participate in this study.

## Author contributions

PK: Writing – original draft, Writing – review & editing, Conceptualization, Data curation, Formal analysis. NA: Writing – original draft, Writing – review & editing, Data curation, Formal analysis. MO: Writing – original draft, Writing – review & editing, Data curation, Formal analysis. AM: Writing – original draft, Writing – review & editing, Data curation, Formal analysis. LP: Writing – original draft, Writing – review & editing. NZ: Writing – review & editing, Writing – original draft. SH: Writing – original draft, Writing – review & editing, Conceptualization, Data curation, Formal analysis, Methodology, Project administration, Supervision.
